# Community engagement and dementia risk: time-to-event analyses from a national cohort study

**DOI:** 10.1136/jech-2019-213029

**Published:** 2019-10-29

**Authors:** Daisy Fancourt, Andrew Steptoe, Dorina Cadar

**Affiliations:** Department of Behavioural Science and Health, University College London Research Department of Epidemiology and Public Health, London, UK

**Keywords:** dementia, ageing, social activities, psychosocial factors, cognition

## Abstract

**Background:**

There is increasing interest in the potential health benefits of referring older adults to engage in community leisure activities (‘social prescribing’) to help promote healthy cognitive ageing. However, it remains unclear whether beneficial effects of community engagement are independent of the well-known protective effects of broader structural, functional and subjective social factors.

**Methods:**

We analysed data from 9550 adults aged 50+ from the English Longitudinal Study of Ageing, with baseline from 2004 to 2005. We assessed associations between different types of community engagement and dementia incidence over a 12-year period. Specifically, we used Cox proportional hazards models, competing risk regressions models, and modified Fine and Gray subdistribution hazards models while controlling for all identified demographic, health-related, and social covariates.

**Results:**

Community cultural engagement (eg, visiting museums, galleries, the theatre) was associated with a lower hazard of developing dementia in older age independent of demographic, health-related and a broad range of social factors, using all three statistical approaches (fully adjusted Cox models: HR 0.58, 95% CI 0.41 to 0.80). Community group engagement (eg, attending clubs or societies) was only associated with dementia prior to adjustment for social factors. Results were robust to sensitivity analyses considering reverse causality, over-adjustment and baseline cognitive function.

**Conclusion:**

It is not just social factors that are associated with reduced risk of dementia onset, but community engagement may also be protective, particularly when relating to cultural activities. These findings are of relevance when considering the current interest in social prescribing to support healthy ageing.

## Introduction

There is increasing interest in the potential health benefits of referring individuals to participate in community leisure activities (often referred to as ‘social prescribing’).^1–3^ In some countries community ‘link workers’ or ‘navigators’ are working alongside clinicians to identify suitable community activities.^4^ In others, prescriptions for community services are being generated through linking patients’ electronic health records with community-resource databases or through community health workers undertaking structured assessments of the social needs of patients.^5^ One area of growing interest is whether social prescribing could help to promote healthy cognitive ageing. Activities such as visiting museums and taking part in community groups have been associated with slower cognitive decline and lower risk of dementia among adults.^6–8^ Studies involving indices of multiple types of activity have shown that the mental, physical and social components of these activities are key to these associations,^9 10^ including through (i) providing cognitive stimulation and thereby contributing to cognitive reserve,^11^ (ii) supporting in cultivation of skills and interests, which can improve cognitive performance,^12^ (iii) helping in emotion regulation and dopamine release which is associated with cognitive flexibility,^8 13^ and (iv) reducing stress levels, depression and sedentary behaviours, all of which are associated with a lower risk of dementia incidence.^14–16^

However, it remains unclear whether the beneficial effects of community engagement are independent of broader social factors. Participation in community activities is inherently social, and there is a broad literature on the cognitive benefits of structural, functional and subjective aspects of social interaction.^17^ Indeed, several studies have shown how factors such as being married and living with somebody are protective against the development of dementia.^18^ Increased risk of cognitive impairment has also been found among adults who are socially isolated,^19 20^ while positive emotional support has been associated with better cognitive ageing.^21^ Further, lower cognitive function, faster cognitive decline and higher risk of dementia incidence have been found among individuals who are lonely.^22 23^ Despite this clear literature on social factors and cognition, previous studies on community engagement have not fully controlled for the potential confounding role of structural, functional or subjective social interaction in analyses of other community activities. It is vital to ascertain whether community engagement does confer additional benefits above and beyond other social interaction to identify whether social prescribing for older adults could have benefits for cognitive ageing. Furthermore, previous studies have not taken into account the competing risk of death in analyses of dementia risk. Given that many older adults die with dementia, ignoring death within analyses can lead to overestimation of the risk of dementia.^24^

Consequently, this study aimed to explore the independent associations between community engagement (both community cultural engagement and community group engagement) and the risk of dementia incidence over a 12-year period, while controlling for a wide range of social confounders and accounting for the competing risk of death in older adults.

## Methods

### Participants

Participants were drawn from the English Longitudinal Study of Ageing; a nationally representative cohort study of adults aged 50+.^25^ Sample members are drawn from the Health Survey for England, which uses a multistage stratified probability sampling design based on postcode sectors. Baseline was wave 2 (2004/2005), and participants were assessed biennially until wave 8 (2016–2017). 8780 participants who formed the core sample for wave 2 were included, and additionally we included participants who entered the study in wave 4 as part of a refreshment sample (n=1525). This provided an overall sample of 10 305 participants, of whom 9610 provided complete data on variables of relevance to this study. We excluded those who already had dementia at baseline (n=60), which provided an analytic sample of 9550.

### Measures

We explored two types of community engagement. ‘Community cultural engagement’ assessed receptive engagement with cultural sites and was measured by asking participants how often they visited (1) a museum, art gallery or exhibition, or (2) the theatre, concert or opera and combining responses from an index of less than once a year, once or two times per year, or every few months or more (Cronbach’s alpha 0.74). ‘Community group engagement’ assessed participation in groups, clubs or societies and was measured by using an index assessing how frequently participants attended (1) meetings relating to a common interest such as a political party, trade union or environmental group, (2) tenants group, resident group, or neighbourhood watch, (3) church or other religious group, (4) charitable association, (5) education class, arts or music group evening class, (6) social club or (7) any other organisation, club or society. The frequency of participation over the past year was categorised into an index of less than once a year, once or two times per year, or every few months or more (Cronbach’s alpha 0.69).

Assessing dementia is recognised as being challenging, particularly in cohort studies, because self-reports of dementia diagnoses can under-report prevalence. Consequently, there is a growing trend in dementia research to use composite indices of self-reported dementia diagnosis and cognitive status.^26–29^ In this study, we used an algorithm that combines self-reported or informant-reported physician diagnosis of dementia or Alzheimer disease or an informant-reported score above the threshold of 3.38 on the 16-question IQCODE (Informant Questionnaire on Cognitive Decline in the Elderly).^26^ This questionnaire is administered to an informant (eg, a family member or a caregiver), who can evaluate the changes in the everyday cognitive function for the past 2 years or more. Each item in IQCODE is scored from 1 (much improved) to 5 (much worse). Previous validations of this approach have shown both high specificity (0.84) and sensitivity (0.82),^26^ suggesting that it can provide useful credible data on dementia incidence. This particular index has also been previously used in a number of studies of risk factors for dementia.^26–29^ Out of 893 dementia cases reported in the paper, 733 (82%) were self-reported clinician diagnoses, and 160 cases (18%) were ascertained via IQCODE score. Of those diagnosed via IQCODE, a further 91 were also diagnosed by a clinician during the study period, leaving just 69 cases (7.7%) that were only ascertained via IQCODE.

Covariates were selected through directed acyclic graphs (DAGs) included demographic covariates (age, sex, educational attainment, wealth, employment status), health covariates (depression, eyesight, hearing, cardiovascular conditions, physical activity) and social covariates (marital status, living status, social contact, social network size, perceived loneliness, perceived positive social support, perceived negative social support). These are explained in full in online supplementary material.

10.1136/jech-2019-213029.supp1 Supplementary data

### Statistical analyses

To assess incidence rates of dementia over the entire follow-up period, we first computed incidence by frequency of social activity per 1000 person-years. We then used three different types of time-to-event analyses to analyse the relationship between social activity and dementia incidence. First, we calculated Cox proportional hazards models with age as the underlying time variable, providing HRs and 95% CIs. Survival time was calculated using participants’ baseline age at study entry until either the age they were found to be experiencing dementia, the midpoint between the wave where dementia was first ascertained and the previous wave where it was not (if a precise date was unavailable), or the end of the study period (the last wave before dropout or the final interview date of wave 8 in 2017). All assumptions, including proportionate hazards, were met.

Second, in order to account for the competing risk of death, we conducted two sets of competing risk models. The first of these was the competing risk regression model, with mortality data provided from the official records from the National Health Service central register (which covered the entire study recruitment period) for all participants who provided consent for data linkage, with a follow-up to latest available data in 2013. The results are shown in online supplementary table 1. The second competing risk model used a modification of the Fine and Gray Subdistribution Hazards model^30^ previously used in dementia analyses,^27 31^ in order to try and reduce survivor bias (whereby individuals who die are censored from the study under the assumption that death is unrelated to the probability of developing dementia). This approach uses a propensity model based on well-known predictor variables for dementia to estimate the probability that individuals had dementia so that deaths with a high probability of having dementia are reclassified as the main event and deaths with a low probability of dementia are maintained as the competing risk event, as described elsewhere.^27^

For all analyses, model 1 was unadjusted, model 2 was adjusted for demographic covariates, model 3 was additionally adjusted for health-related covariates and model 4 was additionally adjusted for a wide range of social covariates. All analyses were weighted to account for differential non-response and to ensure the sample remained representative of the general population. All analyses met all model assumptions. A monotonic trend was computed by considering each exposure as a continuous variable rather than categorical. As only 6.8% of the sample was missing data on covariates and as our analyses for the Subdistribution Hazards models involved generating predictions of responses for individuals who had died; we did not undertake multiple imputations. However, details on the included versus the missing sample are shown in online supplementary table 2. Changes in HRs due to the inclusion of covariates were calculated using the equation (HR (E + C + X) – HR (E + C)) / (1 - HR (E + C))* 100, where HR=hazard ratio, E=exposure, C=covariates already included in model, and X=additional covariates included in new model.

We additionally conducted several sensitivity analyses. First, in order to explore whether participants with early-stage dementia might have altered patterns of community engagement that could bias results, we excluded participants who developed dementia in the first 2 years following baseline (94 cases). Second, we identified through our DAG that several of the confounders in our analyses could be argued to lie on the causal pathway, in which case adjusting for them would not be appropriate. This applied to depressive symptoms cardiovascular disease, physical activity, loneliness, negative and positive social support and size of social network. As it could be debated both ways as to whether these factors do lie on the causal pathway or are merely confounders, we therefore ran analyses that excluded these factors to assess if results were comparable. Third, we considered whether certain factors were potential moderators by including them as interaction terms within the models. This was applied to age, gender, marital status, loneliness, wealth, educational attainment, or employment status. Given that dementia diagnosis prior to age 65 is rare, we also repeated analyses for those aged 65 and over. We also considered whether BMI or other chronic illness additional to cardiovascular disease (such as arthritis, cancer, chronic obstructive pulmonary disease (COPD), Parkinson’s disease or any psychiatric conditions) might confound associations by additionally controlling for these in our analyses. In order to ascertain whether associations were merely a function of people with better cognitive function at baseline engaging more in community activities (as has been suggested in previous studies^32^), we additionally (1) adjusted for baseline cognition and also (2) restricted the sample to those with unimpaired cognition at baseline (top three quartiles). Cognition was measured as an average of standardised scores of memory, executive function, processing speed and orientation in time using validated measures.^33^ Finally, in order to assess whether intellectual activities confounded the relationship, we additionally adjusted for whether respondents read a daily newspaper. Results from sensitivity analyses are provided in online supplementary material. Data are available from the UK Data Service. All analyses were conducted in Stata SE, V.14 (StataCorp).

## Results

Participants had a mean age of 65.2 (SD 9.2, range 50–99) and were 54.3% female. 68.4% engaged socially with family and friends once a week or more, 26.1% engaged in cultural activities every few months or more, and 20.8% engaged in community groups every few months or more. There was a relatively low correlation between community cultural engagement and community group engagement (r=0.33, p<0.001). Both activities showed low correlations with other social activities (see online supplementary table 3). Over the 12-year follow-up period, 429 participants (4.5%) developed dementia. Demographics by dementia outcome are shown in table 1.

**Table 1:** Demographic characteristics of the sample

	Mean (SD)/%	Develops dementia (n=429)	Did not develop dementia within study period (n=9121)	P value
Demographics				
Age	65.2 (9.2)	74.2 (8.2)	64.8 (9.0)	**<**0.001
Gender, female	45.7%	40.3%	46.0%	0.022
Educational attainment				**<**0.001
No qualification	39.7%	55.0%	39.0%	
Education to age 16	17.8%	14.0%	17.9%	
Education to age 18	28.5%	22.1%	28.8%	
Degree	14.1%	8.9%	14.3%	
Working full-time/part-time	36.6%	8.2%	38.0%	**<**0.001
Wealth (bottom quintile)	19.6%	28.2%	19.1%	**<**0.001
Health				
Depressive symptoms score	1.54 (1.96)	2.16 (2.18)	1.51 (1.94)	**<**0.001
Poor eyesight	3.1%	6.5%	3.0%	**<**0.001
Poor hearing	4.6%	10.3%	4.3%	**<**0.001
Cardiovascular condition	40.7%	43.8%	40.6%	0.18
Low levels of physical activity	8.2%	19.8%	7.7%	**<**0.001
Social factors				
Married/living with partner	69.2%	57.8%	69.7%	**<**0.001
Living alone	25.0%	33.1%	24.6%	**<**0.001
Social contact				**<**0.001
Less than once a month	17.3%	26.8%	16.9%	
Once or two times per month	14.3%	13.1%	14.3%	
Once or two times per week or more	68.4%	60.1%	68.8%	
Lonely much of the time in past week	13.3%	21.9%	12.9%	**<**0.001
Number of close friends				**<**0.001
0	25.9%	36.6%	25.4%	
1–2	28.8%	24.7%	29.0%	
3–5	30.3%	22.8%	30.6%	
6+	15.1%	15.9%	15.1%	
Positive social support (score denoting the highest possible positive social support)	19.9%	14.2%	20.2%	0.002
Negative social support (score denoting the highest possible negative social support)	19.1%	30.8%	18.6%	**<**0.001
Community engagement				
Community cultural engagement				**<**0.001
Less than once a year	50.9%	66.7%	50.1%	
Once or two times per year	23.1%	18.4%	23.3%	
Every few months or more	26.1%	14.9%	26.6%	
Community group engagement				0.37
Less than once a year	68.1%	70.9%	68.0%	
Once or two times per year	11.1%	11.0%	11.1%	
Every few months or more	20.8%	18.2%	20.9%	

### Time to dementia

**Figure 1:**
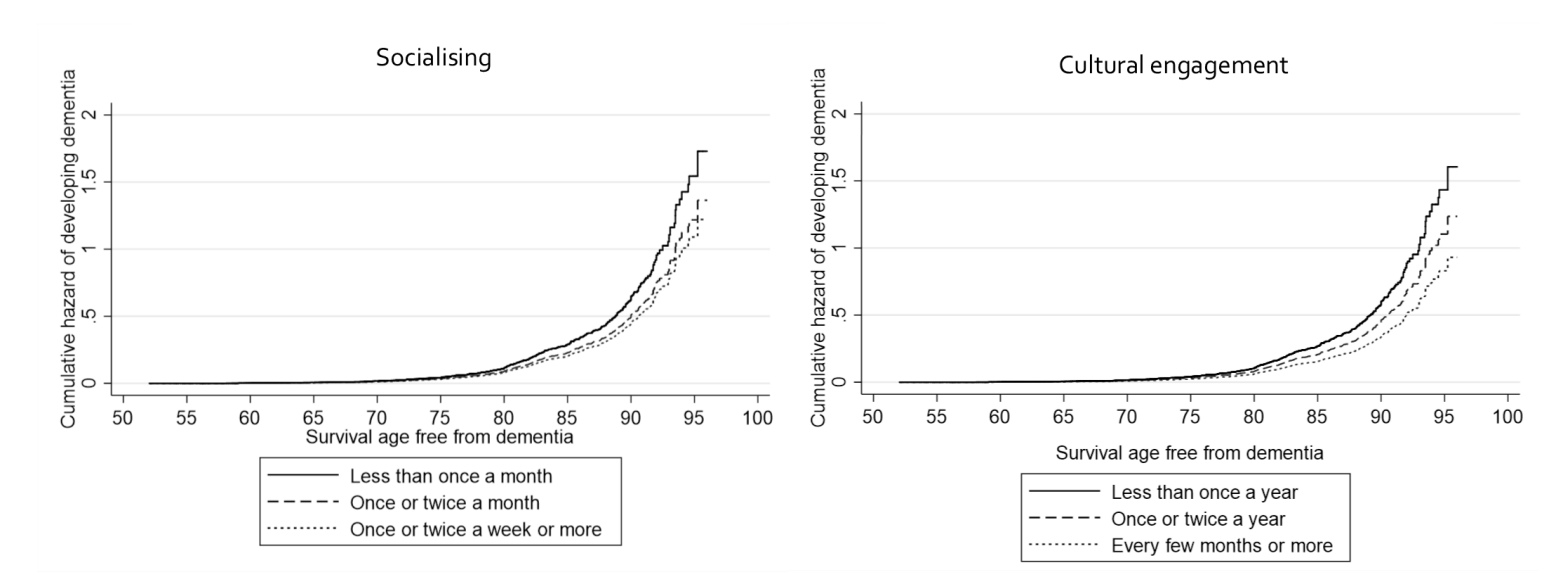
Graphs of Cox proportional hazards regression models showing the cumulative hazard function for social and cultural engagement (both entered simultaneously into the model so mutually adjusted).

Community cultural engagement every few months or more was associated with a 47% lower hazard of developing dementia when accounting for all identified demographic and health-related factors (HR 0.53, 95% CI 0.38 to 0.73), and a 43% lower hazard of developing dementia when additionally adjusting for all identified social factors (HR 0.57, 95% CI 0.41 to 0.80). This suggests that social factors accounted for around 9% of the association between community cultural engagement and dementia. Community group engagement was not associated with a lower hazard in any of the models (table 2). For comparative purposes, in the fully adjusted model, socialising once a week or more was associated with a 29% lower hazard (HR 0.71, 95% CI 0.52 to 0.98), and living with somebody was associated with a 42% lower hazard (HR 0.58, 95% CI 0.60 to 1.30). Figure 1 presents the cumulative hazard estimates for community engagement and socialising in relation to dementia incidence by age.

**Table 2:** Incidence and HRs of dementia by level of community engagement

n=9550; 75 789 person-years Dementia cases: 429	No (cases/censored)	Person-years	HRs (95% CI)	P for trend
Model 1				
Community cultural engagement				
Less than once a year	292/5264	8.0 (7.1–9.0)	1 (Ref)	**<**0.001
Once or two times per year	75/2358	4.1 (3.3–5.2)	**0.65 (0.50 to** **0.84**)	
Every few months or more	58/2622	2.7 (2.1–3.6)	**0.46 (0.34 to** **0.61**)	
Community group engagement				
Less than once a year	307/7064	6.1 (5.5–6.9)	1 (Ref)	0.56
Once or two times per year	45/1112	5.1 (3.8–6.9)	0.88 (0.65 to 1.20)	
Every few months or more	72/2068	4.3 (3.4–5.4)	0.95 (0.72 to 1.24)	
Model 2				
Community cultural engagement				
Less than once a year			1 (Ref)	**<**0.001
Once or two times per year			**0.66 (0.50 to** **0.86**)	
Every few months or more			**0.47 (0.34 to** **0.63**)	
Community group engagement				
Less than once a year			1 (Ref)	0.61
Once or two times per year			0.90 (0.65 to 1.23)	
Every few months or more			0.95 (0.72 to 1.25)	
Model 3				
Community cultural engagement				
Less than once a year			1 (Ref)	**<**0.001
Once or two times per year			**0.71 (0.54 to** **0.94**)	
Every few months or more			**0.53 (0.38 to** **0.73**)	
Community group engagement				
Less than once a year			1 (Ref)	0.87
Once or two times per year			0.95 (0.68 to 1.31)	
Every few months or more			0.99 (0.75 to 1.30)	
Model 4				
Community cultural engagement				
Less than once a year			1 (Ref)	**<**0.001
Once or two times per year			0.77 (0.59 to 1.02)	
Every few months or more			**0.57 (0.41 to** **0.80**)	
Community group engagement				
Less than once a year			1 (Ref)	0.70
Once or two times per year			1.05 (0.76 to 1.47)	
Every few months or more			1.05 (0.79 to 1.41)	

Community cultural engagement and group engagement were entered simultaneously into the models so are mutually adjusted. Model 1 unadjusted. Model 2 adjusted for demographic covariates (age, sex, educational attainment, wealth, employment status). Model 3 additionally adjusted for health covariates (depression, eyesight, hearing, cardiovascular conditions, physical activity). Model 4 additionally adjusted for social covariates (marital status, living status, social contact, social network size, perceived loneliness, perceived positive social support, perceived negative social support).

### Time to dementia or death

When taking account of the competing risk of death and adjusting for demographic and health-related covariates, community cultural engagement every few months or more was also associated with a 39% lower hazard of developing dementia (HR 0.61, 95% CI 0.44 to 0.84). This reduced to a 35% lower hazard (10% decrease) when additionally adjusting for social factors (HR 0.65, 95% CI 0.47 to 0.90). Community group engagement was not associated with a lower hazard in any of the models (see online supplementary table 1).

### Time to dementia or death with a high probability of dementia

When further taking account of participants who died with a high probability of dementia and adjusting for demographic and health-related factors, community cultural engagement every few months or more was associated with a 62% lower hazard of developing dementia (HR 0.38, 95% CI 0.29 to 0.50). This reduced to a 56% lower hazard (10% reduction) when additionally adjusting for social factors (HR 0.44, 95% CI 0.33 to 0.57). Community group engagement once or two times per year was associated with a 31% lower hazard when just accounting for demographic and health-related factors, but this was attenuated when additionally considering social factors (table 3). For comparative purposes, in the fully adjusted model, socialising once a week or more was associated with a 43% lower hazard (HR 0.57, 95% CI 0.46 to 0.70).

**Table 3:** Incidence and adjusted sub-HRs of community engagement controlling for competing risk of death

n=9550; 76 876 person-years Dementia cases: 893	Adjusted sub-HRs (95% CI)	P for trend
Model 1		
Community cultural engagement		
Less than once a year	1 (Ref)	**<**0.001
Once or two times per year	**0.30 (0.24 to** **0.38**)	
Every few months or more	**0.23 (0.18 to** **0.30**)	
Community group engagement		
Less than once a year	1 (Ref)	**<**0.001
Once or two times per year	**0.54 (0.41 to** **0.70**)	
Every few months or more	**0.61 (0.49 to** **0.76**)	
Model 2		
Community cultural engagement		
Less than once a year	1 (Ref)	**<**0.001
Once or two times per year	**0.37 (0.29 to** **0.47**)	
Every few months or more	**0.33 (0.25 to** **0.43**)	
Community group engagement		
Less than once a year	1 (Ref)	0.003
Once or two times per year	**0.64 (0.50 to** **0.84**)	
Every few months or more	**0.79 (0.63 to** **1.00**)	
Model 3		
Community cultural engagement		
Less than once a year	1 (Ref)	**<**0.001
Once or two times per year	**0.41 (0.32 to** **0.52**)	
Every few months or more	**0.38 (0.29 to** **0.50**)	
Community group engagement		
Less than once a year	1 (Ref)	0.012
Once or two times per year	**0.69 (0.53 to** **0.89**)	
Every few months or more	0.82 (0.65 to 1.04)	
Model 4		
Community cultural engagement		
Less than once a year	1 (Ref)	**<**0.001
Once or two times per year	**0.46 (0.36 to** **0.59**)	
Every few months or more	**0.44 (0.33 to** **0.57**)	
Community group engagement		
Less than once a year	1 (Ref)	0.30
Once or two times per year	0.81 (0.62 to 1.07)	
Every few months or more	0.94 (0.74 to 1.20)	

Community cultural engagement and group engagement were entered simultaneously into the models so are mutually adjusted. Model 1 unadjusted. Model 2 adjusted for demographic covariates (age, sex, educational attainment, wealth, employment status). Model 3 additionally adjusted for health covariates (depression, eyesight, hearing, cardiovascular conditions, physical activity). Model 4 additionally adjusted for social covariates (marital status, living status, social contact, social network size, perceived loneliness, perceived positive social support, perceived negative social support).

### Sensitivity analyses

Omitting participants who developed dementia in the first 2 years following baseline did not lead to any attenuation (online supplementary table 4). Only including covariates that did not lie on the causal pathway (so providing less adjusted models) led to consistent but predictably stronger results, including significant associations for community group engagement (online supplementary table 5). Similarly, we found no evidence of moderation by age, gender, marital status, loneliness, wealth, educational attainment, or employment status. When restricting our sample to those aged 65+ results were maintained and were stronger (online supplementary table 6). When additionally controlling for BMI and the presence of other chronic illness, results were maintained for more frequency participation (online supplementary table 7). Finally, controlling for baseline cognition strengthened these associations, suggesting that community engagement is not simply a proxy of higher cognitive status at baseline (online supplementary table 8), and when restricting the sample to those without cognitive impairment results were materially unaffected (online supplementary table 9). Additionally, adjusting for reading a daily newspaper did not affect results (online supplementary table 10).

## Discussion

Overall, this study explored whether community engagement was longitudinally associated with dementia risk in adults aged 50+. Community cultural engagement (every few months or more) was consistently associated with a lower risk of dementia, independent of demographic and health-related confounders and a wide range of social factors and even when taking into account the competing risk of death. Protective associations were not seen consistently for community group engagement, particularly when accounting for other social factors.

Our main finding was that protective associations between community cultural engagement and dementia incidence are maintained independently of wider structural, functional and subjective social factors such as marital status, loneliness, social network size and social support. Social factors explain only around 9%–10% of the overall association. These findings suggest that it is not merely the case that those who engage with cultural activities or with friends and family have higher levels of social engagement, or that community engagement has protective associations merely by compensating for a perceived or actual deficit in social interactions. Instead, our results suggest that aspects of community engagement have cognitive benefits independent of other social factors. This has implications for social prescribing for older adults as it suggests that even among individuals who are not lonely or isolated, encouraging community engagement could potentially be beneficial for reducing the risk of dementia.

However, we only found protective associations for certain types of activities: community cultural engagement but not community group engagement. This supports the results from a previous smaller-scale case-control study that found that cultural engagement from the age of 30 was associated with a lower risk of developing dementia, but group engagement had inconsistent associations.^34^ It is of note that we did see protective associations for community group engagement using the better-powered Fine and Gray models, but these were attenuated when considering social factors. This could imply that social interactions do explain whatever association might exist between community group engagement and dementia risk. Another possible explanation for why we did not see such strong results for community group engagement is that there can be an overlap with activities not carried out for leisure but rather for work, which could lead to feelings of stress or burden.^35^ Indeed, our index included attendance at socially responsible groups such as political groups, tenant’s associations and neighbourhood watch groups alongside more pleasure-oriented groups. Relatedly, it is notable that there was relative heterogeneity in the types of community group activities, some of which may have greater levels of cognitive stimulation and social activity than others. However, as the English Longitudinal Study of Ageing asks about the overall frequency of participation in these activities rather than frequency by group type, we were unable to split the index to consider subgroup associations. Therefore, the consideration of more specific types of community groups and also the potential moderating effect of enjoyment of group engagement could be promising avenues for future research. This is especially important given that our results suggest that certain types of community engagement (such as cultural engagement) may be more appropriate for clinicians to include within social prescribing referrals that have the aim of supporting cognitive ageing among older adults than others.

This study has several limitations. As dementia is challenging to diagnose, the number of dementia cases in our population is likely underestimated. However, we used a validated measure of dementia that combines cognitive testing and self-reported physician diagnosis, used the Fine and Gray method to consider death with probable dementia, and considered the competing risk of death. Notably, our findings were stable across all three statistical approaches. We also had biennial measurement and a 12-year follow-up period. As a result, we applied the best available methods to the dataset. Second, as the number of dementia cases was relatively small, we were also unable to explore associations with specific types of dementia (eg, vascular dementia, mixed dementia or Alzheimer’s disease). This remains for future studies. Third, although social and cultural behaviours have been shown to be relatively consistent in older age,^8^ we remain unsure how previous lifetime social and community engagement might have affected dementia risk. This is particularly relevant when considering that early signs of dementia can develop decades prior to dementia onset, and could affect social behaviours. Our analyses were consistent even when controlling for baseline cognition and when excluding those in the bottom quartile of cognition at baseline, but this remains to be explored further in future studies. Additionally, we focused specifically on visiting cultural venues, but future studies could consider whether results are consistently found across broader types of activities such as carnivals, festivals and participatory arts. Future research could also explore whether accessibility of cultural venues within the community moderates the results found here. Finally, we cannot exclude the risk of unidentified confounding, so although we controlled for a rich set of covariates, causality cannot be assumed.

Overall, this study suggests that it is not just social factors that are associated with a reduced risk of dementia onset, but that community engagement may also be protective, particularly when relating to cultural activities. There has been increasing interest in the UK and internationally in social prescribing schemes, which involve referring individuals with or at risk of illness to community activities, including visiting cultural sites.^3^ The results presented here suggest that there could be a value to social prescribing among older adults to promote healthy cognitive ageing. Future studies are, however, encouraged to understand in more detail how and why community cultural engagement may help to reduce the risk of dementia incidence.

**What is already known on this subject**

- There is a broad literature on the cognitive benefits of structural, functional and subjective aspects of social interaction. Social interaction is known to build cognitive reserve and help to reduce the risk of dementia onset in older age. However, it remains unclear whether community engagement could have protective associations with dementia independent of broader social factors.

**What this study adds**

- This study shows that community cultural engagement is associated with a lower risk of dementia in older adults independent of a wide range of social factors, and is also the first study to show this association is independent of the competing risk of death in older adults. This is important given the increasing interest in the potential health benefits of referring older adults to participate in community leisure activities (‘social prescribing’) to help promote healthy cognitive ageing.
