# Supplementary material for: Community engagement and dementia risk: time-to-event analyses from a national cohort study
Source: J Epidemiol Community Health. 2019 Oct 29;74(1):71–7. doi: 10.1136/jech-2019-213029 (PMC6929705; doi:10.1136/jech-2019-213029)
Supplement: Supplementary data [file jech-2019-213029supp001.pdf]

Supplementary Material

Covariates

Identified demographic covariates included age (as a continuous variable), sex, educational attainment (no formal qualifications, GCE/O-levels/education to age 16, A-levels/education to age 18, university degree or higher), wealth (calculated as net non-pension wealth in quintile categories [30]), and employment status (working full or part-time vs retired/not working). Health-related covariates included depression, eyesight or hearing impairment, the presence of any cardiovascular conditions (including angina, hypertension, diabetes, heart disease, diabetes, previous heart attack or previous stroke), and physical activity behaviour (whether participants took part in either mild, moderate or vigorous activity on a weekly basis or more). We also included a number of covariates covering psychosocial factors such as marital status (married/cohabiting vs single/divorced/widowed), living status (whether living alone or with others), frequency of social contact with friends, relatives or children (less than once a month, once or twice a month, or once or twice a week or more), size of social network (0, 1-2, 3-5 or 6+ close friends), perceived loneliness (measured using the question "how often do you feel lonely" with responses of never/hardly ever, some of the time, or often), perceived positive social support (measured using the questions "how much do your friends really understand the way you feel about things", "how much can you rely on them if you have a serious problem", and "how much can you open up to them if you need to talk about your worries" with responses a lot, some, a little, not at all), and perceived negative social support (measured using the questions "how much do your friends criticise you", "how much do they let you down when you are counting on them" and "how much do they get on your nerves" with responses a lot, some, a little, not at all).

Supplementary Table 1: Incidence and Hazard Ratios for community engagementwith competing risk of death

**N=9,550; 76,876 person-years**<br>**Dementia cases: 429**<br>**Deaths: 1,195**	Model 1	Model 2	Model 3	Model 4
**Cultural engagement**				
**Less than once a year**	1 [Ref]	1 [Ref]	1 [Ref]	1 [Ref]
**Once or twice a year**	**0.70 (0.53-0.91)**	0.78 (0.56-0.95)	0.79 (0.60-1.03)	0.84 (0.63-1.11)
**Every few months or more**	**0.52 (0.39-0.70)**	**0.55 (0.40-0.75)**	**0.61 (0.44-0.84)**	**0.65 (0.47-0.90)**
**Community group engagement**				
**Less than once a year**	1 [Ref]	1 [Ref]	1 [Ref]	1 [Ref]
**Once or twice a year**	0.97 (0.71-1.32)	0.99 (0.72-1.36)	1.05 (0.76-1.45)	1.14 (0.83-1.58)
**Every few months or more**	1.01 (0.77-1.32)	1.04 (0.79-1.37)	1.08 (0.82-1.43)	1.13 (0.85-1.52)

Community cultural engagement and group engagement were entered simultaneously into the model so are mutually adjusted. Results are fully adjusted for demographic covariates (age, sex, educational attainment, wealth, employment status), health covariates (depression, eyesight, hearing, cardiovascular conditions, physical activity), and social covariates (marital status, living status, social contact, social network size, perceived loneliness, perceived positive social support, perceived negative social support).

Supplementary Table 2: Comparison of included to missing data

	Included (n=9,550)	Missing (n=694)	p
Age	65.2 (9.2)	67.6 (13.8)	**<.001**
Gender, female	45.7%	53.6%	**<.001**
Educational attainment			**.043**
No qualification	39.7%	45.1%	
Education to age 16	17.8%	16.9%	
Education to age 18	28.5%	25.2%	
Degree	14.1%	12.8%	
Working full-/part-time	36.6%	35.5%	.54
Depression score	1.54 (1.96)	1.74 (2.05)	**.009**
Poor eyesight	3.1%	4.9%	**.01**
Poor hearing	4.6%	7.2%	**.002**
Cardiovascular condition	40.7%	47.0%	**.001**

Sedentary lifestyle	8.2%	16.9%	**<.001**
Married/living with partner	69.2%	75.7%	**<.001**
Live alone	25.0%	21.2%	**.025**
Lonely	13.3%	14.8%	.24
Number of close friends			**<.001**
0	25.9%	38.2%	
1-2	28.8%	26.5%	
3-5	30.3%	22.8%	
6+	15.1%	12.5%	

Supplementary Table 3: Pearson's correlation coefficients between community engagement and social factors

	Community cultural engagement	Community group engagement
Married/living with partner	0.11	0.06
Live alone	-0.08	-0.04
Social contact	0.22	0.19
Lonely	-0.12	-0.07
Positive social support	0.31	0.23
Negative social support	-0.27	-0.20
Number of close friends	0.29	0.26

All significant p<.001

Supplementary Table 4: Time-to-event analyses omitting participants who developed dementia within 2 years of baseline

**Fully-adjusted Hazard Ratios (95% CI) per Model**		
**N=7,549**<br>**Dementia cases (Cox): 331**<br>**Dementia cases (F&G): 799**<br>**Deaths (for F&G analysis): 731**	Cox analysis (fully-adjusted)	Modified Fine & Grey analysis (fully-adjusted)
**Cultural engagement**		
**Less than once a year**	1 [Ref]	1 [Ref]
**Once or twice a year**	0.84 (0.62-1.14)	**0.46 (0.36-0.59)**
**Every few months or more**	**0.52 (0.37-0.75)**	**0.39 (0.29-0.52)**
**Community group engagement**		
**Less than once a year**	1 [Ref]	1 [Ref]
**Once or twice a year**	1.09 (0.76-1.57)	0.80 (0.60-1.07)
**Every few months or more**	1.19 (0.87-1.62)	0.98 (0.76-1.26)

Community cultural engagement and group engagement were entered simultaneously into the model so are mutually adjusted. Results are fully adjusted for demographic covariates (age, sex, educational attainment, wealth, employment status), health covariates (depression, eyesight, hearing, cardiovascular conditions, physical activity), and social covariates (marital status, living status, social network size, social contact, perceived loneliness, perceived positive social support, perceived negative social support).

Supplementary Table 5: Time-to-event analyses only controlling for covariates that could not lie on the causal pathway

**Adjusted Hazard Ratios (95% CI) per Model**		
**Dementia cases (Cox): 429**<br>**Dementia cases (F&G): 864**<br>**Deaths (F&G): 760**	Cox analysis	Modified Fine & Grey analysis
**Cultural engagement**		
**Less than once a year**	1 [Ref]	1 [Ref]
**Once or twice a year**	**0.66 (0.50-0.86)**	**0.37 (0.29-0.47)**
**Every few months or more**	**0.47 (0.35-0.65)**	**0.33 (0.26-0.44)**
**Community group engagement**		
**Less than once a year**	1 [Ref]	1 [Ref]
**Once or twice a year**	0.92 (0.67-1.27)	**0.65 (0.50-0.85)**
**Every few months or more**	0.97 (0.73-1.27)	**0.79 (0.63-1.00)**

Community cultural engagement and group engagement were entered simultaneously into the model so are mutually adjusted. Results are adjusted just for age, sex, educational attainment, employment status, wealth, eyesight, hearing and cardiovascular disease.

Supplementary Table 6: Time-to-event analyses restricted to those aged 65+

**Adjusted Hazard Ratios (95% CI) per Model**		
**N=4,563**<br>**Dementia cases (Cox): 804**<br>**Dementia cases (F&G):788**<br>**Deaths (F&G): 585**	Cox analysis (fully-adjusted)	Modified Fine & Grey analysis (fully-adjusted)
**Cultural engagement**		
**Less than once a year**	1 [Ref]	1 [Ref]
**Once or twice a year**	**0.46 (0.36-0.60)**	**0.45 (0.35-0.59)**
**Every few months or more**	**0.41 (0.31-0.56)**	**0.46 (0.34-0.61)**
**Community group engagement**		
**Less than once a year**	1 [Ref]	1 [Ref]

**Once or twice a year**	0.75 (0.56-1.01)	0.77 (0.58-1.04)
**Every few months or more**	0.86 (0.66-1.10)	0.92 (0.72-1.18)

Community cultural engagement and group engagement were entered simultaneously into the models so are mutually adjusted. Results are fully adjusted for demographic covariates (age, sex, educational attainment, wealth, employment status), health covariates (depression, eyesight, hearing, cardiovascular conditions, physical activity), and social covariates (marital status, living status, social contact, social network size, perceived loneliness, perceived positive social support, perceived negative social support).

Supplementary Table 7: Time-to-event analyses additionally adjusting for other chronic illnesses & BMI

**Adjusted Hazard Ratios (95% CI) per Model**		
**Dementia cases (Cox): 804**<br>**Dementia cases (F&G):893**<br>**Deaths (F&G): 585**	Cox analysis (fully-adjusted)	Modified Fine & Grey analysis (fully-adjusted)
**Cultural engagement**		
**Less than once a year**	1 [Ref]	1 [Ref]
**Once or twice a year**	0.77 (0.58-1.02)	**0.46 (0.36-0.59)**
**Every few months or more**	**0.57 (0.41-0.79)**	**0.43 (0.33-0.57)**
**Community group engagement**		
**Less than once a year**	1 [Ref]	1 [Ref]
**Once or twice a year**	1.06 (0.76-1.47)	0.81 (0.62-1.07)
**Every few months or more**	1.06 (0.79-1.42)	0.95 (0.74-1.21)

Community cultural engagement and group engagement were entered simultaneously into the models so are mutually adjusted. Results are fully adjusted for demographic covariates (age, sex, educational attainment, wealth, employment status), health covariates (depression, eyesight, hearing, cardiovascular conditions, physical activity, chronic illness, BMI), and social covariates (marital status, living status, social contact, social network size, perceived loneliness, perceived positive social support, perceived negative social support).

Supplementary Table 8: Time-to-event analyses additionally controlling for baseline cognition

**Adjusted Hazard Ratios (95% CI) per Model**		
**Dementia cases (Cox): 429**<br>**Dementia cases (F&G): 864**<br>**Deaths (F&G): 760**	Cox analysis (fully-adjusted)	Modified Fine & Grey analysis (fully-adjusted)
**Cultural engagement**		
**Less than once a year**	1 [Ref]	1 [Ref]
**Once or twice a year**	0.86 (0.64-1.15)	**0.48 (0.37-0.62)**
**Every few months or more**	**0.59 (0.41-0.84)**	**0.44 (0.33-0.58)**
**Community group engagement**		
**Less than once a year**	1 [Ref]	1 [Ref]
**Once or twice a year**	1.14 (0.79-1.65)	0.87 (0.65-1.16)
**Every few months or more**	1.25 (0.90-1.72)	1.05 (0.81-1.36)

Community cultural engagement and group engagement were entered simultaneously into the models so are mutually adjusted. Results are fully adjusted for demographic covariates (age, sex, educational attainment, wealth, employment status), health covariates (depression, eyesight, hearing, cardiovascular conditions, physical activity), social covariates (marital status, living status, social contact, social network size, perceived loneliness, perceived positive social support, perceived negative social support), and standardised baseline cognition.

Supplementary Table 9: Time-to-event analyses excluding those in the bottom quartile of cognition at baseline

**Adjusted Hazard Ratios (95% CI) per Model**		
**N=7,721**<br>**Dementia cases (Cox): 201**<br>**Dementia cases (F&G): 422**<br>**Deaths (F&G): 442**	Cox analysis (fully-adjusted)	Modified Fine & Grey analysis (fully-adjusted)
**Cultural engagement**		
**Less than once a year**	1 [Ref]	1 [Ref]
**Once or twice a year**	**0.57 (0.37-0.88)**	**0.33 (0.23-0.48)**
**Every few months or more**	**0.50 (0.32-0.78)**	**0.35 (0.24-0.50)**
**Community group engagement**		
**Less than once a year**	1 [Ref]	1 [Ref]
**Once or twice a year**	1.18 (0.74-1.88)	0.83 (0.56-1.21)
**Every few months or more**	1.20 (0.79-1.83)	0.98 (0.70-1.37)

Community cultural engagement and group engagement were entered simultaneously into the models so are mutually adjusted. Results are fully adjusted for demographic covariates (age, sex, educational attainment, wealth, employment status), health covariates (depression, eyesight, hearing, cardiovascular conditions, physical activity), and social covariates (marital status, living status, social contact, social network size, perceived loneliness, perceived positive social support, perceived negative social support).

Supplementary Table 10: Time-to-event analyses additionally controlling for reading

**Adjusted Hazard Ratios (95% CI) per Model**		
**Dementia cases (Cox): 429**<br>**Dementia cases (F&G): 864**<br>**Deaths (F&G): 760**	Cox analysis (fully-adjusted)	Modified Fine & Grey analysis (fully-adjusted)
**Cultural engagement**		
**Less than once a year**	1 [Ref]	1 [Ref]
**Once or twice a year**	0.78 (0.59-1.03)	**0.84 (0.64-1.11)**
**Every few months or more**	**0.58 (0.42-0.80)**	**0.65 (0.47-0.91)**
**Community group engagement**		
**Less than once a year**	1 [Ref]	1 [Ref]
**Once or twice a year**	1.06 (0.76-1.47)	1.14 (0.82-1.58)
**Every few months or more**	1.05 (0.79-1.41)	1.14 (0.85-1.52)

Community cultural engagement and group engagement were entered simultaneously into the models so are mutually adjusted. Results are fully adjusted for demographic covariates (age, sex, educational attainment, wealth, employment status), health covariates (depression, eyesight, hearing, cardiovascular conditions, physical activity), social covariates (marital status, living status, social contact, social network size, perceived loneliness, perceived positive social support, perceived negative social support), and reading a daily newspaper.
